# Intraoperative visualization of morphological patterns of the thoracic duct by subcutaneous inguinal injection of indocyanine green in esophagectomy for esophageal cancer

**DOI:** 10.1002/ags3.12594

**Published:** 2022-06-22

**Authors:** Shigeo Tokumaru, Masato Kitazawa, Satoshi Nakamura, Makoto Koyama, Yuji Soejima

**Affiliations:** ^1^ Division of Gastroenterological, Hepato‐Biliary‐Pancreatic, Transplantation and Pediatric Surgery, Department of Surgery Shinshu University School of Medicine Nagano Japan

**Keywords:** esophageal cancer, indocyanine green, near‐infrared fluorescence imaging, prone position, thoracic duct, thoracoscopic esophagectomy

## Abstract

To prevent chylothorax after esophageal cancer surgery, it is important to recognize morphological patterns of the thoracic duct intraoperatively. The present study aimed to evaluate the safety and usefulness of near‐infrared (NIR) fluorescence imaging with subcutaneous inguinal injection of indocyanine green (SII‐ICG) to detect the thoracic duct during thoracoscopic esophagectomy for esophageal cancer. Patients (n = 16) who underwent thoracoscopic esophagectomy in the prone position with SII‐ICG at Shinshu University Hospital between June 2020 and January 2022 were enrolled in the present study and retrospectively reviewed. Immediately prior to thoracoscopic esophagectomy, we injected 0.2–0.5 mg/kg ICG into the subcutaneous tissue in the bilateral inguinal region. The identification rate of the thoracic duct was 93.8% (n = 15), and the success rate of fluorescence using SII‐ICG was 87.5% (n = 14). The visible thoracic ducts had four patterns: a typical pattern in 50% (n = 8), duplication pattern in 18.8% (n = 3), branching pattern in 12.5% (n = 2), and plexiform pattern in 12.5% (n = 2). In all cases, ICG fluorescence did not disappear and was visible during the thoracic surgery. No SII‐ICG‐related complications were observed. Intraoperative NIR fluorescence imaging of the thoracic duct using SII‐ICG is a simple and safe method with very high detection sensitivity. This method can be a powerful tool for avoiding thoracic duct injuries during esophageal cancer surgery.

## Graphical abstract

Intraoperative near‐infrared fluorescence imaging of the thoracic duct using SII‐ICG is a simple and safe method with very high detection sensitivity. We believe that our study makes a significant contribution to the literature because this method can be a powerful tool for avoiding thoracic duct injuries during esophageal cancer surgery.

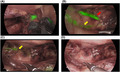

## 1 INTRODUCTION

Chylothorax is a complication that occurs in about 1%–2% of esophagectomies, and it is a refractory complication that can induce pneumonia and sepsis.^1^, ^2^, ^3^ Resection of the thoracic duct is considered a risk factor of metastatic recurrence to more organs.^4^ On the other hand, Matsuda et al reported that thoracic duct resection during esophagectomy could increase the efficiency of lymph node dissection and improve the prognosis in early esophageal cancer.^5^ Either way, intraoperative visualization of the detailed anatomy of the thoracic duct can reduce postoperative complications.

It has been reported that the typical pattern of the thoracic duct is observed in only about 50% of people.^6^, ^7^ Various variations of the thoracic duct pattern have been reported, including complete left‐sided pattern, complete right‐sided pattern, proximal and distal duplication, and plexiform variation.^8^ In addition, it is known that there are lymphatic vessels in the thoracic cavity that flow directly into the thoracic duct.^7^ Therefore, it is important to recognize the detailed location and pattern of the thoracic duct in each case to prevent accidental ligation of the thoracic duct due to injury and postoperative chylothorax.

Near‐infrared (NIR) fluorescence imaging with inguinal lymph node injection of indocyanine green (ICG) has been reported to be useful for detecting thoracic duct injury sites and treating chylothorax.^9^ We hypothesized that NIR fluorescence imaging with subcutaneous inguinal injection of indocyanine green (SII‐ICG) would be useful for visualizing the thoracic duct anatomy during esophagectomy. The aim of the present study was to evaluate the safety and usefulness of NIR fluorescence imaging with SII‐ICG to detect the thoracic duct during thoracoscopic esophagectomy for esophageal cancer.

Since this imaging procedure differs from previous reports, in that it involves injection into the subcutaneous fat rather than the inguinal lymph nodes, we report its details and effects here as a novel method.

## 2 PATIENTS AND METHODS

### 2.1 Patients

All consecutive patients (n = 21) who underwent thoracoscopic esophagectomy in the prone position at Shinshu University Hospital between June 2020 and January 2022 were enrolled in the present study and retrospectively reviewed. We excluded patients without SII‐ICG (n = 3), esophageal invasion of hypopharyngeal carcinoma (n = 1), and unable to examine due to setting error (n = 1). We reviewed the medical records and collected the patient characteristics. The identification of the thoracic duct was considered positive when the gastrointestinal surgeon could clearly identify the duct on both the retrospective review of the surgical video and the surgical record.

### 2.2 Surgical technique

In our hospital, patients orally consume a high‐fat diet before surgery to enable easy identification of the thoracic duct during surgery. Approximately 4 h before the start of esophagectomy, patients were administered 20% soy oil (Intralipos Injection 20%; Otsuka Pharmaceutical Factory, Tokushima, Japan). Thoracoscopic esophagectomy was performed in the prone position. Five thoracoscopic ports were used: a 12‐mm port was created at the seventh intercostal space (ICS) on the mid‐axillary line by optical trocar access,^10^ and the remaining four ports were placed at the ninth ICS on the inferior to the scapular tip line, the fifth ICS at the posterior axillary line, and the sixth and third ICS on the mid‐axillary line. The chest cavity was inflated using a carbon dioxide insufflation pressure of 6 mm Hg. The azygos vein was divided at the arch level. The mediastinal pleura was incised at the superior mediastinum. The anatomy of the thoracic duct was visualized. The thoracic duct is usually preserved. After mobilization of the esophagus in the thoracic cavity, the thoracic duct was checked for damage. After the thoracic drain was inserted into the chest cavity, the patient was repositioned in the supine position for abdominal and cervical surgery. In all cases, a feeding jejunal tube was placed through the reconstructive gastric tube. Enteral feeding was started within 48 h after surgery. If there were any findings that suggested chylothorax, we temporarily discontinued enteral feeding.

### 2.3 ICG fluorescence NIR lymphography

We injected 0.2–0.5 mg/kg of ICG (Diagnogreen; Dai‐Ichi Sankyo Pharma, Tokyo, Japan) diluted in 5 mL of physiological solution into the subcutaneous tissue in the bilateral inguinal region immediately before repositioning with ultrasound visualization (Figure S1). The dose of ICG was administered at 0.2 mg/kg based on the report of Yang et al,^12^ but was increased accordingly at the surgeon's discretion. In fact, we have the impression that there is no difference in the sensitivity of ICG at doses of 0.2 to 0.5 mg/kg, and we believe that 0.2 mg/kg is sufficient. The time required to administer the ICG was 1–2 min. The VISERA ELITE II System (Olympus Medical Systems, Tokyo, Japan) or 1688 Advanced Imaging Modalities (AIM) 4 K platform (Stryker Japan K.K., Tokyo, Japan) were used for ICG‐enhanced NIR. The thoracic duct intraoperative identification rate was the primary outcome measure. The secondary outcome was the identification of thoracic duct variants, adverse reactions, pain, iatrogenic lesions, and complications at the injection site.

## 3 RESULTS

### 3.1 Patients' characteristics

Clinicopathological features of the patients are summarized in Table 1. The present study involved 16 patients who underwent esophagectomy with SII‐ICG for esophageal cancer, with a median age of 66 y. A high proportion of patients had adenocarcinomas (n = 5, 31.3%). All patients had advanced cancer, and many received neoadjuvant chemotherapy (n = 14, 87.5%).

**TABLE 1:** Clinicopathological features

Factors	SII‐ICG (n = 16)
Age, year (median (range))	66 (79–22)
Sex, male	14 (87.5)
Body mass index, kg/m^2^ (mean ± SD)	23.4 ± 3.6
ASA‐PS	
≤1	2 (12.5)
2	13 (81.3)
≥3	1 (6.3)
COPD	1 (6.3)
Diabetes	2 (12.5)
Clinical cancer stage^a^	
0–I	0 (0)
II–IV	16 (100)
Pathological cancer stage^a^	
0–I	6 (37.5)
II–IV	10 (62.5)
Histology	
Squamous cell carcinoma	10 (62.5)
Adenocarcinoma	5 (31.3)
Other type	1 (6.3)
Neoadjuvant chemotherapy	
FP	4 (25.0)
DCF	9 (56.3)
Other	1 (6.3)
No	2 (12.5)
Preoperative oral intake of fat	15 (93.8)
Field of lymphadenectomy	
Two‐field	4 (25.0)
Three‐field	12 (75.0)
Thoracic duct dissection	5 (31.3)
Pharyngo‐laryngo‐esophagectomy	1 (6.3)

*Note:* Data are presented as n (%).

Abbreviations: ASA‐PS, American Society of Anesthesiologist Physical Status; COPD, chronic obstructive pulmonary disease; DCF, docetaxel+cisplatin+5‐FU; FP, cisplatin+5‐FU; SII‐ICG, subcutaneous inguinal injection of indocyanine green.

^a^ According to the definition of Japanese classification of esophageal cancer 11th edition.

### 3.2 Surgical outcomes

In the present study the median time for thoracic manipulation was 333.5 (range, 171–405) min (Table 2); the median time from SII‐ICG to the start and the median time to the end of the thoracic operation were 44 (range, 15–93) and 383.5 (range, 221–506) min, respectively (Table 3). Blood loss, complications, and amount of postoperative pleural fluid were similar to those of previous esophagectomies (data not shown). Intraoperative thoracic duct injury in this study was 12.5% (n = 2), both of which were abnormal thoracic ducts passing through the right side of the esophagus. One patient was found to have a minor chylothorax that improved with a low‐fat diet for a few days. The case involved a thoracic duct with a duplication pattern, and both ducts were ligated. No chylothorax was more severe than grade 2 according to the Clavien–Dindo classification (CD).

**TABLE 2:** Surgical outcomes in patients

Factors	SII‐ICG (n = 16)
Thoracoscopic operation time, min (median (range))	333.5 (171–405)
Blood loss (mean ± SD)	238 ± 229
POD1 Thoracic drainage (mean ± SD), ml	430 ± 194
Complications	
Chylothorax^a^	1 (6.3)
Pneumonia^a^	1 (6.3)
Anastomotic leak^a^	1 (6.3)
Recurrent laryngeal nerve palsy^a^	1 (6.3)
CD grade ≥3	7 (43.8)
Thoracic duct identification	15 (93.8)
Thoracic duct fluorescence	14 (87.5)

*Note:* Thoracic drainage is defined as the amount of fluid drained from a thoracic drain. Data are presented as n (%).

Abbreviations: CD, Clavien–Dindo classification; POD, postoperative day.

a CD grade ≥2.

**TABLE 3:** Demographics and clinical data of patients with SII‐CG

	Age	Gender	Site	Histology	NAC	Time^a^ to start	Time^b^ to identify	Time^a^ to finish	Camera System	TD fluorescence	TD recognition	ICG dose Mg (mg/kg)	SII‐ICG‐related complications	TD patterns
1	65	M	Ut	SCC	DCF	46	56	352	Olympus	**Yes**	**Yes**	20 (0.27)	No	Typical
2	79	F	Mt	SCC	FP	46	91	296	Olympus	**Yes**	**Yes**	20 (0.48)	No	**Duplication**
3	79	M	Ae	Adeno	No	30	86	221	Stryker	**Yes**	**Yes**	22 (0.37)	No	**Branching**
4	71	M	Ae	Adeno	No	93	138	264	Olympus	**Yes**	**Yes**	25 (0.34)	No	**Branching**
5	76	M	Lt	SCC	DCF	17	57	344	Stryker	**Yes**	**Yes**	25 (0.37)	No	**Duplication**
6	67	M	Ce	SCC	DCF	15	‐	506	Olympus	No	**Yes**	25 (0.34)	No	Typical
7	59	M	Ut	SCC	FP	37	97	385	Stryker	**Yes**	**Yes**	20 (0.40)	No	**Duplication**
8	22	F	Mt	SCC	DCF	70	112	393	Stryker	**Yes**	**Yes**	20 (0.40)	No	Typical
9	68	M	Mt	SCC	DCF	35	128	421	Stryker	**Yes**	**Yes**	25 (0.39)	No	Typical
10	71	M	Mt	other	DCF	60	119	359	Stryker	**Yes**	**Yes**	25 (0.44)	No	**Plexiform**
11	57	M	Lt	SCC	DCF	60	126	345	Stryker	**Yes**	**Yes**	25 (0.38)	No	Typical
12	57	M	Lt	Adeno	DCF	58	125	461	Stryker	**Yes**	**Yes**	21 (0.40)	No	Typical
13	42	M	Lt	Adeno	DCF	66	199	471	Stryker	**Yes**	**Yes**	25 (0.27)	No	Typical
14	63	M	Mt	SCC	FP	41	‐	424	Stryker	No	No	24 (0.41)	No	Could not identify
15	67	M	Ae	Adeno	FP	42	93	382	Stryker	**Yes**	**Yes**	25 (0.31)	No	Typical
16	50	M	Ut	SCC	DCF	34	172	400	Stryker	**Yes**	**Yes**	25 (0.30)	No	**Plexiform**

Abbreviations: Adeno, adenocarcinoma; DCF, docetaxel+cisplatin+5‐FU; NAC, neoadjuvant chemotherapy; SCC, squamous cell carcinoma; SII‐CG, inguinal injection of indocyanine green; TD, thoracic duct.

^a^ Time from subcutaneous injection of ICG to the start or finish of the thoracic operation in min.

^b^ Time from subcutaneous injection of ICG to the observation of ICG‐induced fluorescence of the thoracic duct.

TD fluorecence and TD recognition fields are bolded for positive results. TD patterns field is bolded where non‐typical anatomy was identified.

### 3.3 NIR fluorescence imaging with SII‐ICG

**FIGURE 1:**
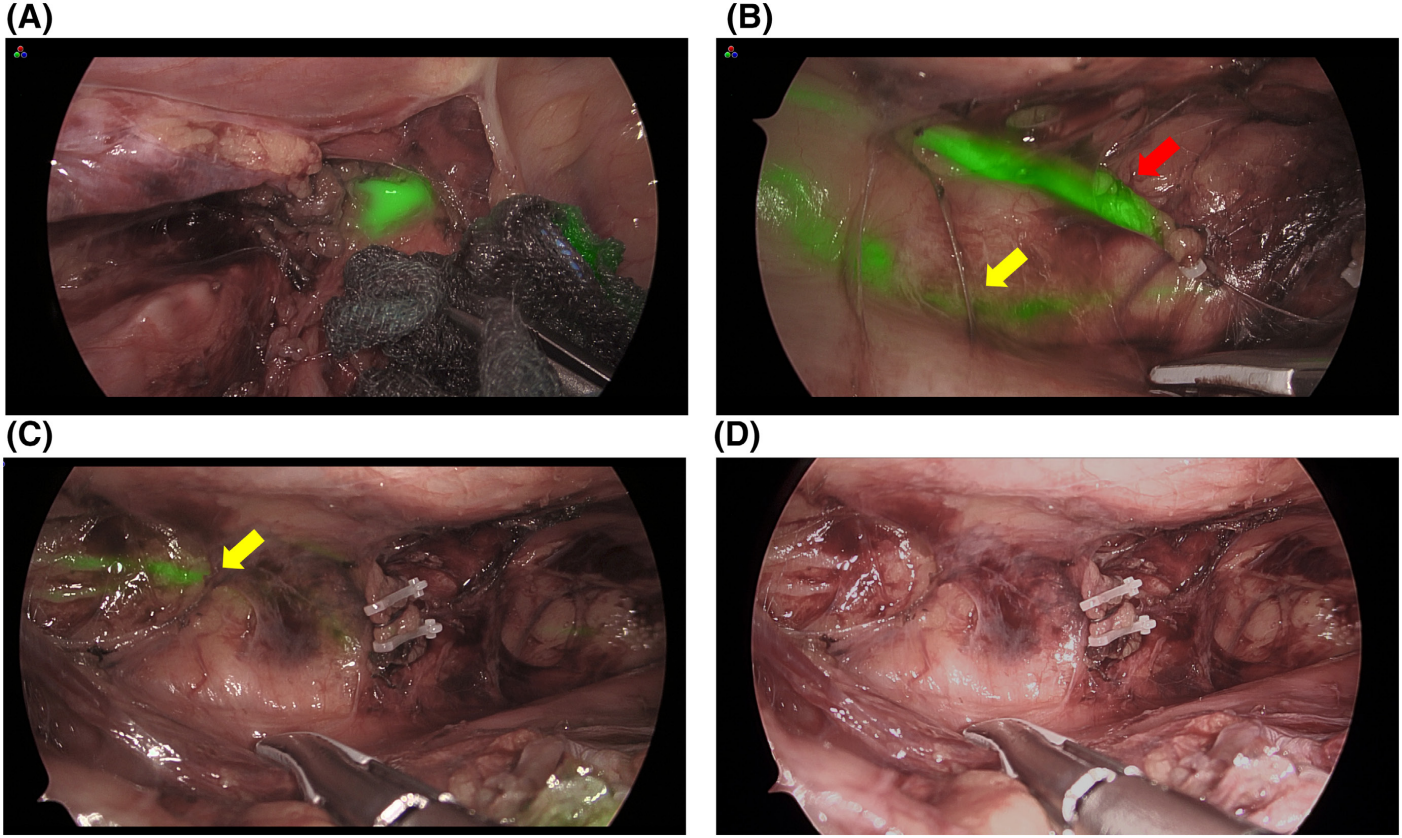
Image of duplication pattern of the thoracic duct. (A) Lymphatic fluid leaking from the abnormal thoracic duct behind the right subclavian artery. (B) Yellow arrow shows typical pattern of the thoracic duct toward the left subclavian vein. Red arrow shows the thoracic duct in an abnormal pattern to the right subclavian vein. (C) The abnormal thoracic duct is securely clipped and resected, checking that no lymphatic fluid leaked from the duct. (D) Observation in normal light. The typical pattern of the thoracic duct was difficult to identify

**FIGURE 2:**
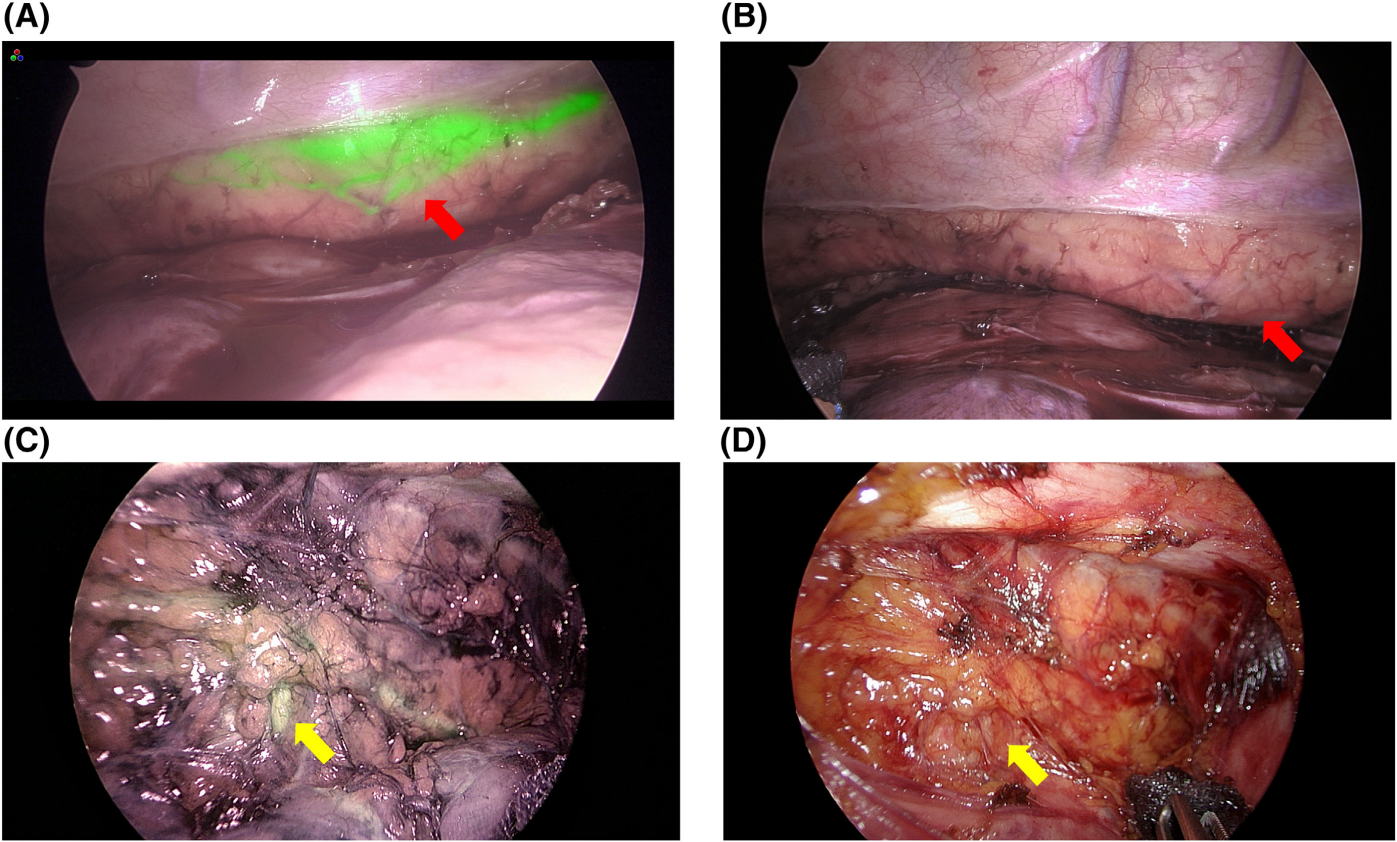
Image of the plexiform pattern and the branching of thoracic duct. (A,B) Red arrow shows the plexiform pattern of the thoracic duct. (A) the plexiform pattern is clearly visualized by indocyanine green (ICG) fluorescence. (B) When near‐infrared (NIR) fluorescence imaging is turned off, the recognition of the plexiform pattern becomes unclear. (C,D) The yellow arrow indicates branching of the thoracic duct in the upper mediastinum. (C) NIR fluorescence imaging. (D) image using normal light

The identification rate of the thoracic duct was 93.8% (n = 15), and the success rate of fluorescence using SII‐ICG was 87.5% (n = 14) (Tables 2 and 3). A case in which the thoracic duct was visible, but no ICG fluorescence was observed, showed direct invasion of the metastatic lymph nodes in the thoracic duct. In another case, in which the thoracic duct was not visible and ICG fluorescence was not observed, we did not find anything that could be the cause. We observed the following patterns of running and branching patterns of the thoracic duct: the typical pattern was observed in 50% of patients (n = 8), with a duplication pattern (Figure 1, Videos S1 and S2) in 18.8% (n = 3), branching patterns (Figure 2A,B) in 12.5% (n = 2), and the plexiform pattern (Figure 2C,D) in 12.5% (n = 2) (Video S3). The median time from SII‐ICG to the observation of ICG‐induced fluorescence of the thoracic duct was 119 min (range, 56–199 min). Even in the patient who was observed in the shortest 56 min, the thoracic duct was well visualized. In all cases, thoracic duct fluorescence continued until the end of the thoracic operation, without additional intraoperative ICG administration. Several slender patients complained of skin discoloration due to ICG after surgery, but it had disappeared in all cases at discharge. No SII‐ICG‐related complications were observed. (Table 3)

## 4 DISCUSSION

We reported that NIR fluorescence imaging with SII‐ICG was useful for detailed intraoperative thoracic duct pattern delineation. It had a high success rate (87.5%) and an adequate duration (Table 3). Recently, Vecchiato et al^11^ reported the usefulness of NIR fluorescence imaging with ICG for thoracoscopic esophagectomy. Before the surgery, ICG was percutaneously injected into the superficial inguinal lymph nodes bilaterally with ultrasound visualization, a step requiring ~10 min.^11^ Barbato et al reported the usefulness of SII‐ICG fluorescence of the thoracic duct during robotic esophagectomy.^12^ That study has suggested that ICG should be administered 14–16 h prior to surgery,^12^ but our results show that intraoperative visualization of the thoracic duct can be achieved by administering ICG immediately prior to surgery. The first advantage of this study is that it used a simple method. The second advantage is that we observed fluorescence of the thoracic duct using ICG for a longer time than the method of injecting it into the lymph nodes. The median time from SII‐ICG to the completion of thoracic operation was 383.5 min (221–506 min) (Table 3). The third advantage of this method is that it can be administered subcutaneously after general anesthesia and does not cause pain to the patient. The fluorescent effect of ICG did not disappear during the operation and was effective for a long time. The first advantage of this study, using NIR fluorescence imaging with ICG, was that the thoracic ducts were clearly distinct from the surrounding tissue and easily recognizable. We visualized the branching and plexiform pattern of the thoracic duct in detail during surgery, which was previously undetected (Figure 2, Videos S3 and S4). This is the first report that morphologic patterns of the thoracic duct can be recognized intraoperatively with the NIR fluorescence method. As in previous reports, no complications were associated with SII‐ICG (Table 3). NIR fluorescence imaging using SII‐ICG was performed after general anesthesia, which was painless. In this study we experienced two cases of intraoperative thoracic duct injury. One case was very skinny and was treated intraoperatively with ICG to prevent leakage from the injured abnormal thoracic duct, but a small amount of chylothorax was observed postoperatively. We suspect that the chylothorax was caused by inadequate treatment of the patient with fragile tissue. In a previous review of 60 cases of thoracoscopic esophagectomy at our hospital, intraoperative thoracic duct injury was 6.7% (n = 4) and postoperative chylothorax was 3.3% (n = 2). Neither the rate of thoracic duct injury nor the incidence of chylothorax differed significantly between patients with and without SII‐ICG. Similarly, there was no significant difference in the thoracic duct identification rate. Major anomalies such as duplicate thoracic ducts were not observed in cases before performing SII‐ICG. NIR fluorescence imaging with SII‐ICG may be useful in identifying thoracic duct anomalies (data not shown).

The benefit of intraoperative NIR fluorescence imaging with ICG is that it can be used in conjunction with conventional methods. Recently, this empirical method involving administering olive oil has been reported to be useful.^13^, ^14^ This method can be used in conjunction with NIR fluorescence imaging with ICG. In this study ICG fluorescence was confirmed with preoperative 20% soy oil oral administration.

Detecting the pattern of the thoracic duct before surgery is useful for preventing intraoperative thoracic duct injury. Lymphangiography^8^ and special magnetic resonance imaging^15^ (MRI) have been used to describe the pattern of the thoracic duct preoperatively. Preoperative lymphangiography is not recommended as a routine procedure because it requires the injection of contrast media into the peripheral lymphatic vessels,^8^ which is technically difficult and highly invasive. MRI is a minimally invasive examination,^15^ and we consider that the combination of intraoperative NIR fluorescence imaging with SII‐ICG and preoperative MRI can be used to determine the pattern of the thoracic duct more accurately.

Intraoperative ligation of the thoracic duct has been reported as a risk factor for chylothorax.^16^ Only one case of minor chylothorax was observed in this study, and minor thoracic duct injury, visible only on NIR fluorescence images, was not detected. NIR fluorescence imaging with ICG provides real‐time visual confirmation of lymphatic fluid leakage from the thoracic duct, which may lead to safer thoracic duct ligation (Figure 2, Video S2). We were concerned that subcutaneous injections of ICG might cause skin problems such as coloration, and in fact several slender patients complained of skin discoloration, which disappeared in all cases upon discharge.

The present study had several limitations. First, it had a retrospective design. Second, it was a single‐center study with a small sample size. Third, two different detection instruments were used, and the difference in instruments may have affected the results. Fourth, preoperative administration of 20% soy oil may have affected the results, but we have not been able to examine its impact. However, there are few reports of NIR fluorescence imaging with SII‐ICG used for monitoring the thoracic duct during long‐period esophagectomy; therefore, our present results are interesting. We were not able to determine the optimal dose of ICG.

## 5 CONCLUSION

Intraoperative NIR fluorescence imaging of the thoracic duct using SII‐ICG is a simple and safe method with very high detection sensitivity, and long‐term fluorescence intensity was maintained for a long time. This method can be a powerful tool for avoiding thoracic duct injuries during esophageal cancer surgery.

## DISCLOSURE

Funding: This research did not receive any specific grants from funding agencies in the public, commercial, or not‐for‐profit sectors.

Conflict of Interest: The authors declare no conflicts of interest for this article.

Author Contributions: S. Tokumaru: study concept and design, acquisition of data, statistical analysis, and drafting of the article. K. Masato: acquisition of data and revision of the article. S. Nakamura: acquisition of data. M. Koyama: acquisition of data. Y. Soejima: study concept and design, study supervision, critical revision of the article.

Ethical Statement: The present study was approved by the Ethics Committee of Shinshu University School of Medicine (Approval No. 5116) and was performed according to the principles of the Declaration of Helsinki. All patients fully understood the study and provided consent to participate in the study. Oral administration of 20% soy oil and subcutaneous injection of ICG are off‐label use, and was used only after approval from Shinshu University Hospital Ethics Committee (Approval No. B0593) and obtaining written consent from the patients.

## Supporting information


Figure S1

Video S1

Video S2

Video S3

Video S4

## Data Availability

Data supporting the findings of this study are available from Shigeo Tokumaru upon reasonable request.
