# Supplementary figures and images for: Intraoperative visualization of morphological patterns of the thoracic duct by subcutaneous inguinal injection of indocyanine green in esophagectomy for esophageal cancer
Source: Ann Gastroenterol Surg. 2022 Jun 22;6(6):873–9. doi: 10.1002/ags3.12594 (PMC9628221; doi:10.1002/ags3.12594)

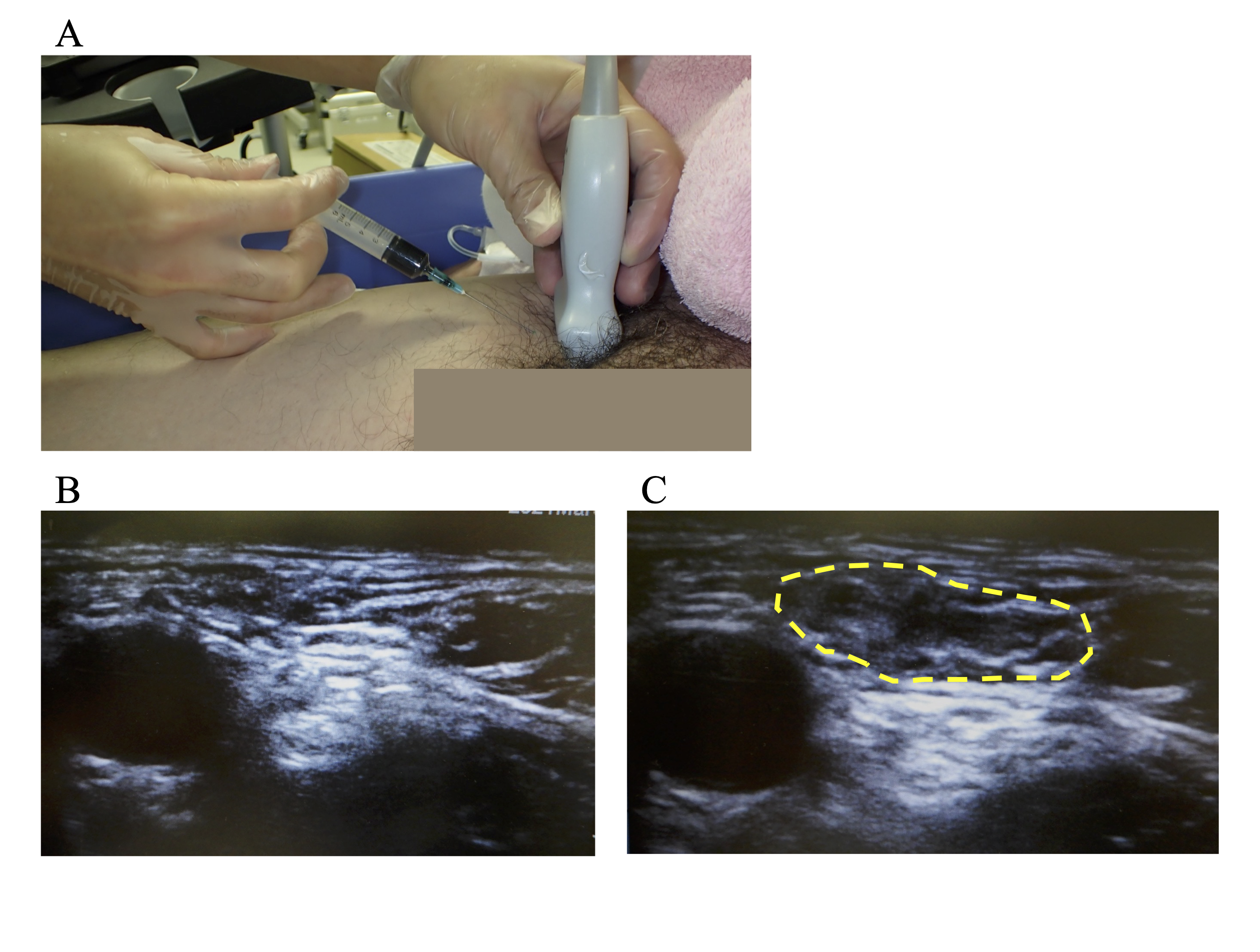

Supplement: Supplementary file 1 — Figure S1 [file AGS3-6-873-s002.tiff]
